# Alcohol consumption and cancer progression: Mechanistic insights, immune dysregulation, and public health implications

**DOI:** 10.1016/j.tranon.2026.102793

**Published:** 2026-04-29

**Authors:** Ravindra Pramod Deshpande, Abhishek Tyagi, Kounosuke Watabe

**Affiliations:** a Department of Biotechnology, Savitribai Phule Pune University, Pune-411057, Maharashtra, India; b Department of Cancer Biology, Wake Forest University School of Medicine, Winston-Salem, NC 27157, USA

**Keywords:** Alcohol consumption, Therapy resistance, Oxidative damage, Immune dysfunction

## Abstract

Alcohol consumption is a modifiable, dose‑dependent risk factor that accelerates cancer initiation, progression, and therapy resistance across breast, liver, colorectal, esophageal, and melanoma cancers. Alcohol metabolism generates acetaldehyde, a genotoxic metabolite that induce DNA damage and impairs repair; drives oxidative stress, hormonal imbalance and promoting a tumor‑supportive microenvironment. These effects are amplified by obesity and insulin resistance, which heighten aggressiveness and blunt treatment response. Alcohol reshapes the tumor immune microenvironment (TIME) and undermines therapies, including immunotherapy. It impairs dendritic cell antigen presentation and co‑stimulation, limits CD4⁺/CD8⁺ priming, and expands immunosuppressive MDSCs and M2‑like TAMs that inhibit cytotoxic T cells and express checkpoint ligands. Concurrent cytokine shifts (TNFα, IL‑6), persistent ROS–NF‑κB signaling, and stromal/vascular remodeling promote immune exclusion and weaken responses to checkpoint blockade, targeted agents, and chemotherapy. Alcohol‑related gut dysbiosis is linked to ICB responsiveness—dampens antitumor immunity. Alcohol increases exosome biogenesis; circulating vesicles enriched in hepatocyte and inflammatory miRNAs (e.g., miR‑122, miR‑192) reprogram immune signaling and metastasis, forming a vesicular axis linking exposure to TIME dysfunction and resistance. Risk is synergistic with tobacco and worsens with aging as detoxification and DNA repair decline. Sex‑specific pharmacokinetics and composition confer vulnerability in women, while socioeconomic barriers delay diagnosis and care. Personalized prevention strategies that incorporate alcohol reduction, smoking cessation, and nutritional support, with emerging biomarkers such as exosomal miRNAs and alcohol‑related metabolites for early detection and risk stratification, are needed to mitigate alcohol‑related carcinogenesis, given the lack of definitive human evidence on alcohol’s independent effects on immunotherapy and chemotherapy efficacy.

## Highlights

• Alcohol is a modifiable, dose-dependent carcinogen linked to cancer initiation, progression, and treatment resistance—especially in breast, liver, colorectal, esophageal, and melanoma cancers.
• Mechanisms include DNA damage (via acetaldehyde), oxidative stress, hormonal disruption (e.g., elevated estrogen), and pro-inflammatory signaling (e.g., NF-κB).
• Risk increases with comorbidities like obesity and insulin resistance, which worsen tumor behavior and reduce treatment response.
• Older adults and women are more vulnerable due to age-related detox decline and sex-specific metabolism, even at moderate intake.
• Public health efforts should prioritize alcohol reduction, smoking cessation, and culturally tailored interventions, with biomarkers aiding early detection and risk stratification.

## Graphical abstract

**Figure:**
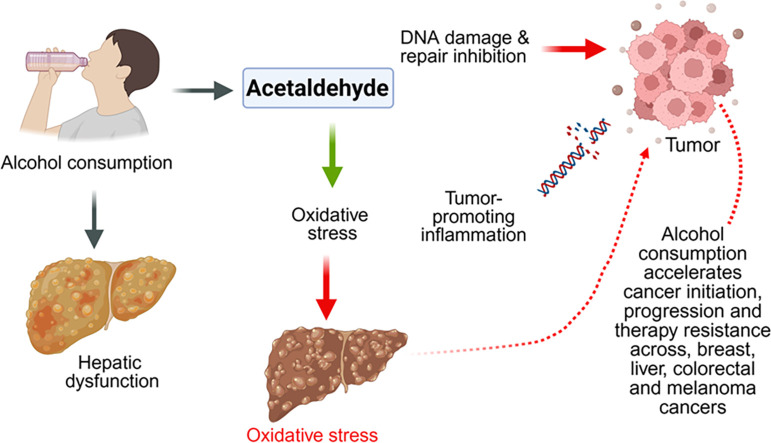

## Background

Alcohol consumption in the U.S. and globally varies significantly across age, gender, income levels, and preferred alcoholic beverage types. According to the 2023 National Survey on Drug Use and Health (NSDUH), 84.9% of U.S. adults aged 18 and older reported lifetime alcohol use, with men (86.6%) slightly more likely than women (83.3%) to consume alcohol [1,2]. Age is known to play a crucial role in the choice of drink with younger adults (18–34) tend to favor beer and spirits, while older adults (55+) lean more toward wine [3,4]. In addition, between 2014 and 2022, households with annual incomes over $91,980 showed the highest increase in purchasing low- and non-alcohol products, rising from 0.6% to 2.5%. In contrast, households earning less than $26,280 showed minimal change. This indicates that higher income groups are more likely to adopt alcohol-reduction behaviors, possibly due to greater health awareness and access to alternatives [5].

Excessive alcohol consumption poses serious health risks, both in the short and long terms. In the short term, it increases the likelihood of injuries from motor vehicle accidents [6], falls, and burns [7], as well as the risk of violence [8], alcohol poisoning, and engaging in risky sexual behaviors that may lead to sexually transmitted infections or unplanned pregnancies [9,10]. Over the long term, chronic alcohol use is strongly linked to liver cirrhosis [11] and several types of cancer, including breast, liver, colon, and esophageal cancers [12]. Therefore, alcohol misuse can lead to immediate injuries and long-term chronic diseases, including liver cirrhosis, cardiovascular issues, and multiple cancers.

Alcohol consumption has long been studied for its complex relationship with cancer risk, with growing evidence highlighting the importance of beverage type and consumption patterns. Among alcoholic beverages, beer and white wine have shown more consistent associations with increased cancer risk compared to red wine. Regular or heavy beer consumption has been linked to elevated risks of colorectal, lung, and esophageal cancers [[13], [14], [15]]. Unlike red wine, beer lacks significant levels of protective polyphenols such as resveratrol that have demonstrated anti-cancer properties, including inhibition of cancer cell proliferation and modulation of immune responses [16,17]. Similarly, white wine intake has been associated with increased risk of skin cancer and melanoma, with relative risks of 1.22 [1.14–1.30] and 1.13 [1.04–1.24], respectively [18,19]. These findings contrast with moderate red wine consumption, which may offer some protective effects due to its richer antioxidant profile. Additionally, Chyou et al., have reported that alcohol consumption—including beer, wine, and spirits—was strongly associated with an increased risk of upper aerodigestive tract cancers among Japanese American men in Hawaii. The risk rose with higher intake, and the association was consistent across all types of alcoholic beverages, suggesting that alcohol itself, rather than specific drink types, contributes significantly to cancer risk in this region of the body [20]. Similarly, a meta-analysis of 27 epidemiological studies found a modest but statistically significant association between alcohol intake and colorectal cancer risk, with beer showing the strongest link (relative risk 1.26), followed by liquor and wine [21]. Therefore, while moderate red wine intake may offer some protective effects due to its antioxidant content, beer and white wine are more consistently linked to increased risks of various cancers, including colorectal and upper aerodigestive tract cancers. These findings highlight the importance of beverage choice and moderation in alcohol consumption for cancer prevention.

Importantly, reports of neutral or potentially protective associations with low-to-moderate alcohol consumption are derived predominantly from observational studies and should be interpreted with caution. Such associations are susceptible to residual confounding by correlated lifestyle and socioeconomic factors, reverse causation due to illness related abstention, and heterogeneity in exposure assessment. Consistent with this, Mendelian randomization analyses and authoritative evaluations do not support a causal protective effect of alcohol consumption on cancer risk or outcomes [22,23].

**Fig. 1:**
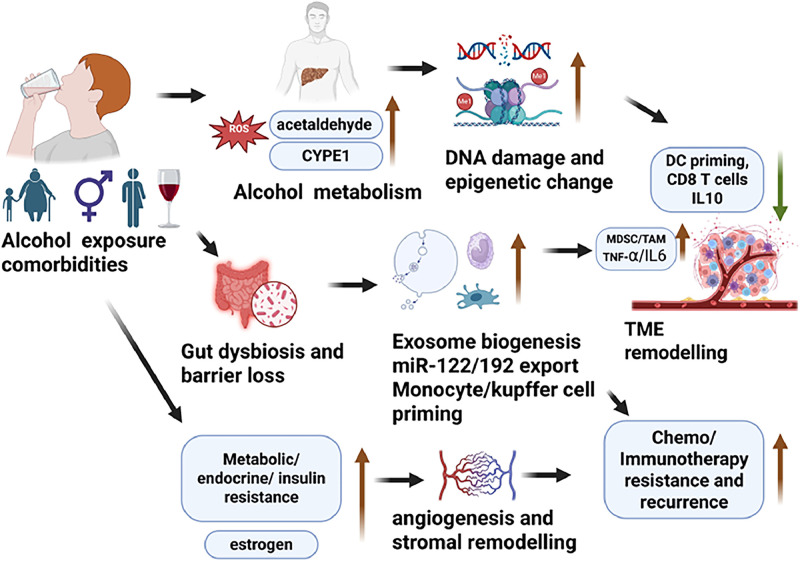
Integrated model illustrating how alcohol exposure contributes to tumor microenvironment (TME) remodeling and therapy resistance. Alcohol metabolism triggers oxidative stress, DNA damage, and epigenetic changes, while associated comorbidities drive gut dysbiosis and metabolic dysfunction. These changes enhance exosome signaling, reshape immune and stromal interactions, and promote angiogenesis. Collectively these processes remodel the tumor microenvironment (TME) and fostering chemo-/immunotherapy resistance and recurrence.

Alcohol consumption has a multifaceted impact on cancer progression, influencing both biological mechanisms and treatment outcomes. A longitudinal study [24] found that approximately 70% of cancer patients continued drinking after diagnosis, with 40–50% exhibiting potentially harmful drinking behaviors. Risky alcohol use was associated with higher anxiety levels, while older age, comorbidities, and depressive symptoms were linked to reduced drinking. This review aims to provide an overview of the direct impact of alcohol consumption on cancer progression and therapy resistance through mechanisms including genotoxic stress, chronic inflammation, and immune dysregulation, while also considering modifying factors such as sex and metabolic comorbidities, including obesity. To integrate mechanistic, immunological, and clinical dimensions of alcohol‑associated cancer progression, we present a conceptual framework (Fig. 1). Fig. 1 provides an integrative conceptual framework connecting alcohol exposure with metabolic and genotoxic stress, tumor immune microenvironment remodeling, and resistance to immunotherapy, targeted therapies, and chemotherapy, thereby outlining the overarching logic of this review.

## Main text

### Alcohol as a risk factor for various cancers

In the following section, we provide an overview of the impact of alcohol consumption on cancer progression and the underlying mechanisms, as reported through the clinical studies (Table 1).

**Table 1:** Impact of alcohol consumption on cancer progression.

Cancer type	Study cohort	No of subjects	Alcohol type	Alcohol dose	Region	Risk Ratio		
and CI	Conclusion	Reference						
Breast	Ivneet Sohi et al.,	8646,779	All types	10 *g*/day	Worldwide	Premenopausal= 1.03 (CI: 1.01–1.06) Postmenopausal= 1.10 (CI: 1.08–1.12)	Alcohol consumption is associated with both pre‐ and postmenopausal breast cancer risk	(26)
	Reiko Suzuki et al.,	229,366	All types	NA	Worldwide	ER+=1.27 (CI: 1.17–1.38) ER-=1.14 (CI: 1.03–1.26)	Alcohol consumption is associated with risk of ER+ and ER- breast cancer	(42)
	Sangah Shin et al.,	74,522						
	All types	NA	Asia	1.75 (CI: 1.33, 2.30)	High alcohol intake was associated with an increased risk	(28)		
	Mary Beth Terry et al.,	4420	All types	NA	USA, Japan	ER+= 0.88 (CI: 0.79–0.98) ER-= 1.23 ( CI: 0.98–1.55)	Alcohol cessation may reduce ER + but not ER- breast cancer risk	(27)
Oral	V Bagnardi et al.,	13,895	All types	Light-moderate and heavy	Worldwide	5.1 (CI: 4.31–6.10)	Alcohol increases risk of cancer of oral cavity	(43)
Esophageal	V Bagnardi et al.,	10,633	All types	Light-moderate and heavy	Worldwide	4.9 (CI: 3.86–6.34)	Alcohol increases risk of cancer of esophageal cancer	(43)
Laryngeal	V Bagnardi et al.,	7059	All types	Light-moderate and heavy	Worldwide	2.6 (CI: 2.19–3.19)	Alcohol increases risk of laryngeal cancer	(43)
Colorectal	Janine Wieser et al.,	11,829,567	All types	10 *g*/day	Worldwide	1.39 ( CI: 1.20–1.59)	Alcohol consumption is rick factor for early-onset CRC (EOCRC)	(35)
	Siyu Huang	5874,483	All types	NA	Worldwide	1.56 (CI: 1.28–1.89)	Early onset colorectal cancer (EOCRC) risk increased with alcohol consumption, especially in men	(34)
Yuwei Li et al.,	33,870	All types	NA	Japan	1.08 (CI: 1.05–1.12)	Potential causal association between alcohol consumption and the risk of CRC among Asians	(37)	
Youngyo Kim	32,846							
	All types	NA	Worldwide	0.87 (CI: 0.78–0.98)	Light prediagnostic alcohol consumption was associated with lower risk of mortality	(36)		
V Bagnardi et al.,	26,932	All types	Light-moderate and heavy	Worldwide	1.4 (CI: 1.25–1.65)	Alcohol increases risk of cancer of colorectal cancer	(43)	
	Sooyoung Cho et al.,	18,522	All types	NA	Korea	1.93 ( CI: 1.17–3.18)	Alcohol consumption was associated with increased colorectal cancer risk among Korean men	(44)
Liver	Shu-Chun Chuang et al.,	5816,371	All types	Ever drinker	Worldwide	1.29 (CI: 1.16–1.42)	One alcoholic drink/day associated with increased risk of liver cancer. Women had higher risk than men at same dose.	(39)
	Hana Park et al.,	15,758	All types	12.5 g/drink	Worldwide	1.41 (CI: 1.1 to 1.6)	Alcohol consumption increases risk of liver cancer in male and female	(38)
Melanoma	Sara Gandini et al.,	10,555	All types	12 g/drink	Worldwide	1.96 (CI: 1.02–3.76)	Alcohol drinking may be moderately associated with increased melanoma risk	(40)
	M Rota et al.,	6251	All types	NA	Worldwide	1.20 ( CI: 1·01–1·44)	Alcohol consumption is positively associated with the risk of cutaneous melanoma	(41)

#### Breast cancer

Alcohol consumption is a well-established modifiable risk factor for breast cancer, with numerous studies confirming a dose-dependent relationship between alcohol intake and increased breast cancer risk [25]. N. Hamajima et al. demonstrated that even low levels of alcohol consumption can elevate risk: one drink per day is associated with a 7–10% increase in breast cancer risk, while consuming two to three drinks daily raises the risk by approximately 20% compared to non-drinkers [26]. Similarly, a meta-analysis by Tsunehisa Nomura et al. confirmed that alcohol significantly raises the risk of developing breast cancer, particularly hormone receptor-positive subtypes, although it found no significant impact on prognosis, recurrence, or survival after diagnosis [27], suggesting alcohol’s role may be more prominent in cancer initiation than progression. However, more recent evidence complicates this view. A systematic review [28] reaffirmed alcohol as a causal factor in breast cancer, with risk increasing even at low levels of consumption (Table 1). Post-diagnosis alcohol use appears to be associated with adverse outcomes, including increased recurrence and mortality, especially among postmenopausal and overweight women [29]. The Pathways Study [30] found no significant association between short-term alcohol consumption around diagnosis and recurrence or mortality overall, though obese women showed a lower risk of all-cause mortality with moderate intake. Notably, alcohol cessation is linked to the reduced risk of estrogen receptor-positive (ER+) breast cancer, but not to the estrogen receptor-negative (ER−) subtypes [29], likely due to alcohol’s influence on estrogen pathways. The absence of a protective effect for ER−negative cancers may reflect reverse causation, missing ER status data, or differences in drinking patterns. Additionally, alcohol use tends to decline during early survivorship, influenced by chemotherapy, financial stress, and changes in physical health [29]. Mechanistically, alcohol contributes to carcinogenesis through several pathways: increased circulating estrogen levels [31], DNA damage via acetaldehyde formation [32,33], oxidative stress [34], and impaired folate absorption [35]. However, more recent studies suggest post-diagnosis alcohol use may worsen outcomes, particularly in postmenopausal and overweight women [28,29], and alcohol cessation may reduce risk for ER+ but not ER− breast cancer [29]. These findings underscore the importance of personalized survivorship care and counseling, considering individual risk profiles, tumor biology, and lifestyle factors when addressing alcohol use in breast cancer patients. Overall, evidence for breast cancer is largely observational, with consistent signals for risk but more heterogeneous findings for prognosis, influenced by confounding, hormone-receptor status, and reverse-causation effects.

#### Colorectal cancer

Alcohol consumption is increasingly recognized as a multifaceted contributor to colorectal cancer (CRC) onset and progression. Huang et al., undertook a rigorous systematic review and meta-analysis of cohort and case–control studies, demonstrating a clear association between higher alcohol intake and elevated risk of early-onset CRC, irrespective of age, sex, or tumor location [36]. Wieser et al., extended these findings by examining the combined lifestyle effects of alcohol and smoking. Their analysis of multiple global studies revealed that alcohol use, especially alongside smoking, heightened the likelihood of early-onset CRC, with risk increasing as consumption rose [37]. In contrast to these risk-focused studies, Kim, Je, and Giovannucci explored survival outcomes, finding that individuals who consumed light to moderate amounts of alcohol before diagnosis experienced more favorable CRC survival compared to non-drinkers, particularly when wine was consumed [38]. Finally, Yuwei Li et al., utilized Mendelian randomization to bridge observational and causal inference, showing that genetic predisposition to alcohol consumption whether measured as ever versus never drinking or volume-based intake was linked to increased CRC risk, independent of confounding [39]. Together, these findings underscore that alcohol consumption not only elevates the risk for early-onset and overall colorectal cancer through both observational and genetic pathways but also modulates survival outcomes, with light to moderate intake potentially associated with improved prognosis. These findings reflect mixed evidence—observational studies and Mendelian randomization consistently support risk elevation, whereas survival benefits with moderate drinking are likely influenced by residual confounding and lifestyle effect modifiers.

#### Liver cancer

In liver cancer, alcohol is a major hepatocarcinogen, contributing to approximately 32–45% of hepatocellular carcinoma (HCC) cases globally, with rising incidence and mortality among young adults. Notably, even low to moderate alcohol consumption significantly increases liver cancer risk, challenging the notion of a safe threshold. In a systematic review and meta-analysis, Park et al. (2020) found that individuals consuming more than low levels of alcohol (≥1 drink/day for females and ≥2 drinks/day for males) had a 41.8% higher risk of liver cancer incidence and a 16.7% higher risk of liver cancer mortality compared to low-level drinkers [40]. Similarly, Chuang et al. reported a clear dose–response relationship, with relative risks rising from 1.08 for 12 g/day (∼1 drink) to 5.20 for 125 g/day and highlighted synergistic effects with hepatitis and diabetes. Moreover, alcohol-related HCC often presents at more advanced stages due to lower surveillance rates, further complicating treatment outcomes and survival [41]. These findings reinforce the need for public health strategies that address alcohol consumption as a modifiable risk factor in liver cancer prevention, which underscores the importance of reducing alcohol consumption as a preventive and management strategy across multiple cancer types, with attention to timing, dose, and individual risk profiles. In contrast to other cancers, liver cancer evidence is strong and consistent, supported by clear dose–response patterns with minimal likelihood of reverse causation.

#### Melanoma

Recent evidence suggests a significant association between alcohol consumption and cutaneous melanoma risk. In a systematic literature review and dose–response meta-analysis, Gandini et al. [42], have analyzed 20 studies and found that individuals with the highest alcohol intake had a 29% increased risk of melanoma compared to those with the lowest intake. The risk rose by 7% for every 10 g/day increase in alcohol consumption, with cumulative intake showing even stronger associations (SRR = 1.96). Similarly, Rota et al. [43] conducted a meta-analysis of 16 studies and reported a 20% increased risk of melanoma among alcohol drinkers compared to non-drinkers, with moderate-to-heavy drinking showing a pooled relative risk of 1.18. The association remained consistent across study designs and was slightly attenuated when adjusted for sun exposure. These findings highlight alcohol consumption as a potential modifiable risk factor for melanoma, warranting further research into its biological mechanisms and public health implications. However, these associations remain modest and susceptible to confounding—particularly sun-exposure-related factors—making interpretation more cautious.

### Mechanisms of alcohol-induced cancer progression

Having established alcohol as a major risk factor across breast, colorectal, liver, and melanoma cancers, the next section explains the underlying biological mechanisms that make these epidemiologic patterns possible. Specifically, we transition from population level associations to molecular pathways—oxidative stress, DNA damage, epigenetic remodeling, and exosome-mediated signaling—that mechanistically link alcohol exposure to tumor initiation and progression. Across cancer sites, acetaldehyde mediated DNA damage and ROS–NF-κB signaling are broadly implicated, whereas endocrine co-drivers (e.g., estrogen) are most relevant to hormone-sensitive tumors such as breast cancer. Likewise, immune remodeling and microbiome shifts are common themes, but the magnitude and clinical implications vary by site.

#### Alcohol induces oxidative stress and DNA damage

Alcohol consumption induces oxidative stress, which plays a critical role in cancer progression through multiple interconnected mechanisms. Ethanol metabolism is reported to lead to the generation of reactive oxygen species (ROS), causing oxidative DNA damage and mutations that initiate oncogenesis [44]. These ROS are also known to activate key signaling pathways such as NF-κB and MAPK, which promote cell proliferation, survival, and inflammation—factors that drive tumor growth and metastasis [45]. Chronic alcohol intake further weakens the body's antioxidant defense systems, intensifying oxidative stress, and facilitating carcinogenesis [46]. Additionally, Gunda Millonig et al., have reported that alcohol-induced oxidative stress disrupts cellular homeostasis by altering gene expression and inducing epigenetic changes that favor malignant transformation in human esophagus [47]. The resulting pro-inflammatory environment supports angiogenesis and enables tumors to evade immune surveillance, further accelerating cancer progression in colorectal [48], hepatocellular carcinoma [49] and breast cancer [49]. Clinical studies summarized by Tsermpini et al. [50] reinforced these mechanistic insights by demonstrating that individuals with chronic alcohol use exhibit elevated biomarkers of oxidative damage—such as malondialdehyde (MDA) and 4-hydroxynonenal (4-HNE)—alongside reduced antioxidant levels, including glutathione and superoxide dismutase. These imbalances are linked to increased disease severity and progression, particularly in liver and gastrointestinal cancers. Thus, oxidative stress markers correlate with tumor aggressiveness and poor prognosis, which suggests their potential utility as clinical biomarkers. Further supporting this notion, Mansoori et al. [51] have reported how ethanol metabolism via alcohol dehydrogenase (ADH), aldehyde dehydrogenase (ALDH), catalase (CAT), and cytochrome P450 enzymes (especially CYP2E1) lead to ROS production. This ROS trigger lipid peroxidation, protein inactivation, and DNA damage, contributing to mitochondrial dysfunction and cell death. Chronic alcohol exposure also alters the expression of genes involved in redox regulation, such as NOX2, SOD, GPX, and GSTs, disrupting antioxidant defenses and promoting sustained oxidative damage. This oxidative imbalance is central to alcohol use disorder (AUD) and is implicated in neurodegeneration and systemic inflammation, both of which are linked to cancer progression [50].

Furthermore, Turner et al. [52] emphasized that alcohol-induced hangovers are associated with systemic inflammation and oxidative stress, which may contribute to the development of several cancers. Specifically, alcohol consumption has been linked to increased risk of colon [53], laryngeal [54], esophageal [55], and oral cancers [56], primarily due to elevated acetaldehyde levels and persistent oxidative damage. These effects are compounded by immune dysregulation and chronic inflammation, creating a microenvironment conducive to tumor initiation and progression.

**Fig. 2:**
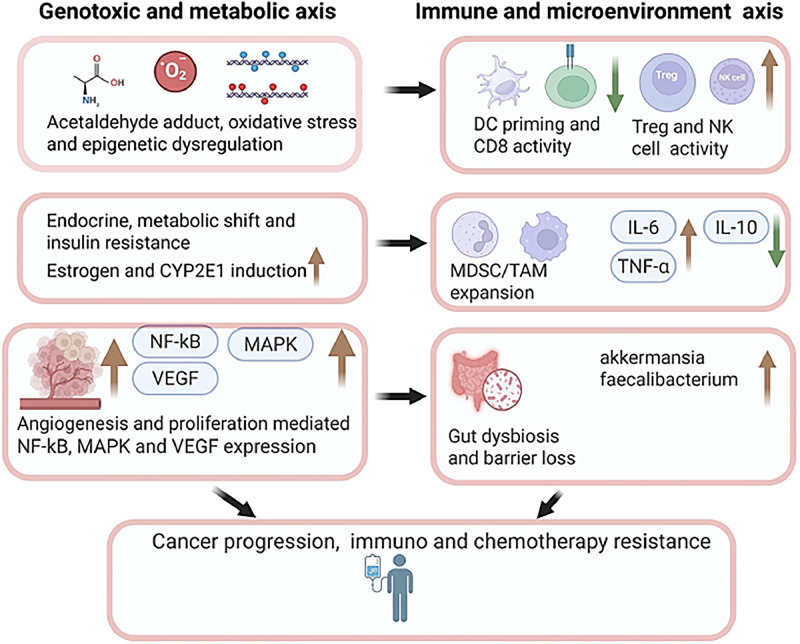
Parallel genotoxic-metabolic and immune-microenvironment axes linking alcohol exposure to tumor progression and therapy resistance. Alcohol-induced genotoxic, metabolic, and immune disruptions act in parallel to reshape the tumor microenvironment, driving cancer progression and resistance to therapy.

In summary, chronic alcohol consumption induces oxidative stress through the generation of reactive oxygen species and disruption of antioxidant defenses, leading to DNA damage, inflammation, and altered gene expression. These changes promote cancer initiation, progression, and metastasis by activating oncogenic pathways, impairing immune surveillance, and fostering a pro-tumor microenvironment (Fig. 2). Fig. 2 serves as an integrative framework linking alcohol‑induced oxidative and endoplasmic reticulum stress with acetaldehyde‑mediated DNA damage and inflammatory signaling, establishing a mechanistic foundation for the immune remodeling and therapeutic resistance described in subsequent sections.

Clinical and genetic studies consistently show that alcohol-related oxidative stress is a key driver of cancer severity and may serve as a valuable target for prevention and therapeutic intervention. Because oxidative and ER stress are potent inducers of exosome biogenesis, these same alcohol-triggered stresses likely drive vesicles output and cargo editing in hepatocytes, setting the stage for miRNA-based immune crosstalk Sundar & [57,58].

#### Alcohol promotes cancer progression by inducing epigenetic modification

Chronic alcohol consumption contributed to cancer progression through multiple molecular and cellular mechanisms. Ethanol metabolism produced acetaldehyde, a carcinogenic metabolite that caused DNA damage, impaired DNA repair, and disrupted methylation patterns, thereby promoting genomic instability and oncogenesis [59]. Alcohol interfered with one-carbon metabolism and reduced folate levels, leading to decreased availability of S-adenosylmethionine (SAM), the primary methyl donor for DNA methylation. This resulted in global hypomethylation and promoter-specific hypermethylation, silencing tumor suppressor genes, and activating oncogenes [60,61]. An epigenome-wide association study identified over 19,000 CpG sites associated with alcohol consumption, many linked to cancer-related genes such as SLC7A11, MCM2, and TRA2B [60]. In breast cancer, alcohol-induced phosphorylation of histone H3 via MSK1 enhanced the expression of Brf1 and RNA polymerase III genes, promoting tumor growth, especially in estrogen receptor-positive cases [61]. These epigenetic changes were further modulated by genetic polymorphisms in enzymes like ADH, ALDH, and MTHFR, which influenced individual susceptibility to alcohol-related carcinogenesis [59].

#### Exosomal miRNAs as mediators of alcohol-induced liver injury and cancer progression

Alcohol exposure does not only injure parenchyma; it also reprograms intercellular communication by increasing hepatocyte exosome biogenesis and exporting immune-active miRNAs, thereby linking exposure to systemic immune dysregulation [57]. In human hepatocytes and in livers from alcohol-associated liver disease, alcohol upregulates exosome machinery (Rabs/VAMPs/syntaxins) and shifts miRNA trafficking—lowering intracellular miR-192/miR-122 while elevating their levels in extracellular vesicles—providing a vesicular route for signaling beyond the liver [57]. Critically, hepatocyte-derived exosomes transfer miR-122 to monocytes/macrophages, suppressing HO-1 and sensitizing them to LPS, which amplifies pro-inflammatory cytokine responses—a direct mechanism by which alcohol drives innate immune activation that can fuel tumor-promoting inflammation ([62]. This vesicle-mediated immune rewiring dovetails with the broader theme that a remodeled tumor immune microenvironment underlies therapeutic resistance, making exosomal miRNAs a mechanistic conduit from alcohol exposure to immune dysfunction and, ultimately, cancer progression and treatment failure [63,64].

Exosomes have emerged as promising biomarkers for alcohol consumption due to their ability to carry molecular signatures reflective of cellular stress and injury. Alcohol exposure was shown to promote exosome biogenesis and release, particularly from hepatocytes, through oxidative stress and modulation of Rab proteins and miRNA expression [57]. These exosomes contained liver-specific miRNAs such as miR-122, which were horizontally transferred to immune cells, suggesting a mechanism for systemic effects of alcohol [62]. In patients with alcoholic hepatitis, the number of circulating exosomes was significantly elevated, and their cargo—especially miR-192, miR-122, and miR-30a—demonstrated strong diagnostic potential for alcohol-induced liver injury [65]. Among these miRNAs, miR-122 is highly liver-specific and has been extensively studied for its dual role as a tumor suppressor and oncogene. Alcohol-induced dysregulation of miR-122 has been linked to hepatocellular carcinoma (HCC), where its downregulation promotes cell proliferation, migration, and invasion by targeting genes such as LMNB2 and G6PD, and is further suppressed epigenetically via DNA methylation mechanisms involving HOTAIR [66]. Similarly, miR-192 has been implicated in various cancers, acting either as a tumor suppressor or oncogene depending on the context. Alcohol exposure modulates miR-192 expression, which influences exosome biogenesis and contributes to cancer-related signaling pathways, making it a potential biomarker for alcohol-associated malignancies [67]. In head and neck squamous cell carcinoma (HNSCC), miR-30a was found to be significantly upregulated in alcohol-associated cases. Its overexpression promoted cellular proliferation, invasion, and resistance to chemotherapy, while targeting tumor suppressor genes such as MEF2D, suggesting its role in alcohol-driven tumorigenesis [68]. Together, exosomes released in response to alcohol consumption serve as non-invasive biomarkers for liver injury and systemic inflammation, and their molecular cargo may also contribute to cancer progression, making them valuable tools for both diagnosis and mechanistic insight. By exporting miR-122/miR-192 in exosomes that reprogram monocytes and Kupffer cells, alcohol creates a proinflammatory, immunosuppressive milieu that aligns with—and helps mechanistically explain—the immune microenvironment defects described in the next section.

### Effects of alcohol on tumor immunology and progression

#### Alcohol consumption impairs innate and adaptive arms of the immune system

While several immune effects of alcohol appear generalizable (e.g., impaired antigen presentation, T‑cell dysfunction), others are site‑ and context‑specific, varying with tumor type, comorbidities, and exposure intensity; we therefore highlight shared mechanisms and note site‑specific differences where evidence permits.

Alcohol consumption profoundly disrupts immune regulation, influencing cancer development and progression through multiple mechanisms. Sloan A. Lewis et al. reported that chronic alcohol intake impairs both innate and adaptive immunity, affecting the function of macrophages, dendritic cells, NK cells, and T and B lymphocytes, thereby weakening tumor surveillance and immune-mediated clearance [69]. These cellular impairments reduce the immune system’s ability to detect and eliminate emerging tumor cells, facilitating immune escape, and cancer progression. In addition to cellular dysfunction, alcohol promotes oxidative stress and acetaldehyde accumulation, which damages DNA and interferes with antigen presentation and immune cell signaling [70]. These effects are compounded by alcohol-induced epigenetic dysregulation, which alters gene expression in immune cells and contributes to immune evasion. Such disruptions impair the efficacy of immunotherapies, particularly immune checkpoint inhibitors, by reducing T cell activation and altering PD-1/PD-L1 signaling pathways [71]. Ascensión Marcos et al. have emphasized alcohol’s systemic pro-tumorigenic role across multiple cancer types—including esophageal, liver, colorectal, breast, lung, and skin cancers—highlighting its broad impact on immune and oncogenic pathways [72]. Therefore, alcohol-induced immune dysfunction compromises tumor surveillance and therapeutic response, underscoring the importance of alcohol moderation in cancer prevention and treatment. Alcohol consumption, particularly chronic and heavy intake, exerts significant and largely detrimental effects on the adaptive immune system, impacting both T cells and B cells. Chronic alcohol exposure leads to a reduction in peripheral T cell numbers, disrupts the balance between CD4+ and CD8+ T cells, and impairs T cell activation and survival, often promoting apoptosis and skewing T cells toward a pro-inflammatory phenotype [73,74], thereby compromising the body's ability to mount effective immune responses against pathogens and tumors. Alcohol also affects regulatory T cells (Tregs), reducing their suppressive function, and contributing to systemic inflammation and immune dysregulation [75]. In B cells, alcohol alters their development and distribution. While bone marrow B cell development remains largely unaffected, alcohol increases transitional B cells (T1) and decreases marginal zone B cells, which are crucial for early immune responses [75]. Additionally, alcohol enhances immunoglobulin production, particularly IgM and IgA, and increases autoantibody levels, contributing to autoimmune pathology and alcohol-related liver damage [76]. These effects occur both directly—through ethanol’s impact on immune cell signaling, metabolism, and survival—and indirectly, via alcohol-induced changes in the gut microbiome, cytokine milieu, and oxidative stress, which further modulate adaptive immune responses [75].

Similarly, Li et al., have reported that alcohol consumption decreased the population of myeloid-derived suppressor cells (MDSCs), which are known to promote tumor immune evasion, and increased infiltration of CD4+ and CD8+ T cells in the tumor microenvironment. This immune reprogramming was associated with reduced liver metastasis, suggesting that alcohol may paradoxically exert protective effects in certain contexts by enhancing anti-tumor immunity [24]. Complementing these findings, a systematic review and meta-analysis [77] showed that alcohol consumption during oncological treatment may impair therapeutic effectiveness and increase toxicity. Specifically, alcohol use during radiotherapy was linked to significantly worse disease-free survival (pooled HR: 2.05; 95% CI: 1.09–3.89), likely due to interference with drug metabolism and increased risk of adverse interactions. These studies underscore the complex and context-dependent role of alcohol in cancer progression, emphasizing the need for personalized guidance on alcohol use during and after cancer treatment. Beyond its direct carcinogenic effects, alcohol also impairs immune function—particularly the development, survival, and activity of T and B lymphocytes—thereby weakening adaptive immunity. This immunosuppression increases susceptibility to infections, autoimmune disorders, and cancer recurrence. In individuals with alcohol use disorders, these immune deficits highlight the importance of moderation and regular immune monitoring, especially in the context of cancer care and survivorship.

Alcohol consumption disrupts cytokine homeostasis, leading to chronic inflammation that plays a central role in cancer progression. Ethanol exposure elevates pro-inflammatory cytokines such as TNF-α, IL-1β, IL-6, and MCP-1, while suppressing anti-inflammatory cytokines like IL-10, fostering a persistent inflammatory state [73]. This imbalance contributes to tissue damage, immune dysfunction, and a tumor-promoting microenvironment. In addition, alcohol promotes NF-κB activation through the accumulation of reactive oxygen species (ROS), further driving chronic inflammation and cancer progression. In hepatocellular carcinoma (HCC), alcohol enhances tumor growth and metastasis by upregulating VEGF and MCP-1, both regulated by ROS and NF-κB signaling [44]. Experimental models demonstrate that alcohol-induced ROS directly activate NF-κB in HepG2 cells, and inhibition of this pathway suppresses tumor growth, angiogenesis, and metastasis. These findings align with earlier reports showing that alcohol modulates NF-κB signaling by altering p65 phosphorylation, depending on exposure duration [78], and contributes to cytokine dysregulation by increasing TNF-α, IL-6, IL-1β, and MCP-1, while reducing IL-10 [73,79]. Additionally, alcohol-induced ROS promotes tumor angiogenesis and proliferation through NF-κB-dependent transcriptional activation of pro-tumorigenic genes [80]. ROS also induces mitochondrial damage, disrupting the electron transport chain and oxidative phosphorylation, which impairs cellular energy metabolism and facilitates tumor cell survival and migration [81]. Together, these studies reveal that alcohol’s impact on NF-κB is both direct, via ROS-mediated activation, and indirect, through altered cytokine profiles and immune cell signaling. This dual mechanism fosters a pro-inflammatory and tumor-promoting microenvironment, particularly in cancers such as liver, colorectal, and breast, where NF-κB plays a central role in immune evasion and angiogenesis.

**Fig. 3:**
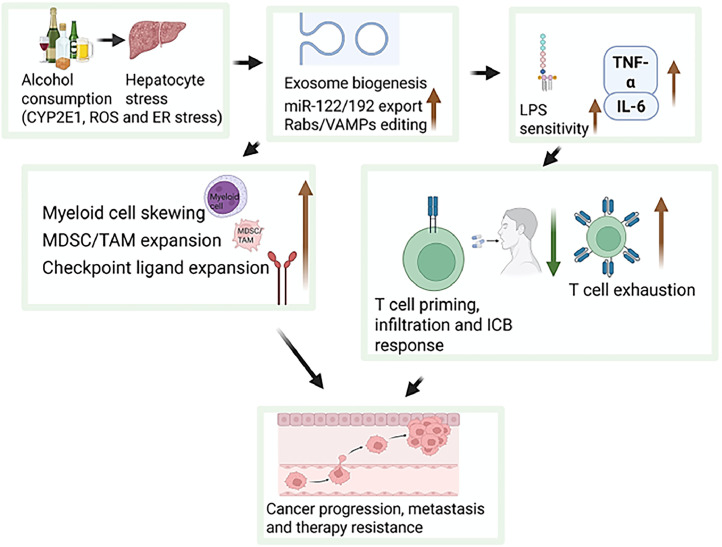
Exosomal miRNA axis linking alcohol exposure to immune dysfunction and therapy resistance during cancer progression. Alcohol-induced exosomal miRNA signaling disrupts immune function by enhancing myeloid skewing, cytokine imbalance, and T-cell exhaustion, collectively promoting tumor progression and therapy resistance.

Chronic alcohol consumption also plays a pivotal role in the development and progression of liver fibrosis, a key precursor to hepatocellular carcinoma. Alcohol-induced liver injury activates hepatic macrophages, including Kupffer cells, which respond to alcohol metabolites and gut-derived endotoxins by releasing pro-inflammatory cytokines such as TNF-α and IFN-γ. These cytokines contribute to hepatocellular damage and stimulate hepatic stellate cells, promoting extracellular matrix deposition and fibrotic remodeling of liver tissue [82]. Additionally, alcohol alters macrophage polarization and signaling pathways—such as Toll-like receptors (TLRs) and MAP kinases—leading to sustained inflammatory responses that exacerbate liver fibrosis [83]. These fibrogenic and inflammatory processes not only compromise liver function but also create a microenvironment conducive to tumor initiation and progression. The central role of macrophages in alcohol-associated liver disease highlights their potential as therapeutic targets for mitigating fibrosis and reducing cancer risk in individuals with chronic alcohol exposure. Alcohol consumption exerts widespread and multifactorial effects on immune regulation, inflammation, and tissue remodeling It impairs both innate and adaptive immunity, disrupts cytokine balance, promotes oxidative stress, and activates oncogenic signaling pathways such as NF-κB. These mechanisms collectively foster a tumor-promoting microenvironment, compromise immunotherapeutic efficacy, and accelerate disease progression—particularly in liver, colorectal, and breast cancer (Fig. 3). Fig. 3 integrates alcohol‑driven alterations across innate and adaptive immune compartments, cytokine imbalance, and inflammatory signaling, summarizing how chronic alcohol exposure reshapes the tumor immune microenvironment to promote tumor progression. Furthermore, alcohol-induced liver fibrosis driven by macrophage activation and stellate cell stimulation underscores its role in organ-specific pathology and cancer risk. These findings reinforce the urgent need for alcohol moderation and targeted interventions in cancer prevention, treatment, and survivorship care.

#### Alcohol mediates immunotherapy resistance

Alcohol consumption has a multifaceted impact on the immune system, contributing to resistance against immune checkpoint inhibitors (ICIs), particularly those targeting PD-1 and PD-L1 pathways. Recent evidence highlights that alcohol consumption impairs the efficacy of anti-PD-1 immunotherapy through multiple mechanisms. Given that alcohol-induced exosomal miRNAs intensify monocyte/macrophage inflammatory signaling, the resultant TIME skew diminishes T-cell priming/function and plausibly contributes to the reduced anti-PD-1 efficacy observed in patients and preclinical models [62,63].

Because alcohol remodels the TIME by impairing T-cell function, dendritic cell priming, cytokine balance, and myeloid cell composition, these immune defects translate directly into reduced responsiveness to immunotherapies. Therefore, the next section examines how alcohol-driven immune remodeling results in diminished efficacy of PD-1/PD-L1 blockade and contributes to broader therapeutic resistance.

One study demonstrated that alcohol disrupts anti-tumor immunity by altering T cell phenotypes and reducing intratumoral T cell infiltration, leading to diminished effector functions and increased regulatory T cell populations. Mechanistically, alcohol interferes with PD-1 signaling and downstream SHP1/2-mediated dephosphorylation, thereby promoting oncogenic pathways such as MAPK and AKT in tumor cells, particularly in lung and bladder cancer models, resulting in enhanced immune evasion and tumor progression [63]. Another study found that alcohol remodels the immunosuppressive tumor microenvironment by targeting myeloid-derived suppressor cells (MDSCs). Chronic alcohol intake reduced MDSC populations in peripheral blood, spleen, and tumor tissues, while increasing CD4+ and CD8+ T cell infiltration. This effect was mediated via suppression of IL-6 and inhibition of the JAK/STAT3 signaling pathway, suggesting that alcohol modulates innate immune signaling to influence tumor immunity [24]. Taken together, preclinical and limited clinical data suggest that alcohol may attenuate responses to anti‑PD‑1/PD‑L1 therapy via T‑cell dysfunction and myeloid remodeling (including IL‑6/JAK–STAT3 signaling); however, definitive reductions in human ICI efficacy attributable to alcohol have not been established.

Mechanistically, alcohol disrupts cytokine signaling and chemokine gradients that regulate immune cell recruitment and activation. Chronic alcohol intake has been shown to upregulate immunosuppressive cytokines such as IL-10 and TGF-β, while suppressing pro-inflammatory mediators like IL-12 and IFN-γ, thereby promoting a tumor-permissive microenvironment [75,84]. This cytokine imbalance hampers antigen presentation and reduces the effectiveness of immune priming, which is critical for checkpoint blockade therapies. Because alcohol‑driven oxidative stress and cytokine dysregulation often coexist with tumor hypoxia, the resulting low‑oxygen environment further suppresses T‑cell activity and impairs checkpoint responses. Preclinical studies indicate that oxygen‑supplying nanomaterials can mitigate hypoxia and enhance T‑cell‑mediated antitumor activity, supporting their exploration as adjuncts to counter hypoxia‑exacerbated immunosuppression in alcohol‑remodeled TIME; prospective human data are needed [85].

Importantly, alcohol impairs dendritic cell (DC) function, which is central to initiating and sustaining anti-tumor immune responses. A study [86] revealed that ethanol exposure inhibits DC antigen presentation and reduces their ability to activate antigen-specific cytotoxic T cells. In female mice, ethanol-treated DCs showed reduced expression of activation markers (CD44, CD69) and decreased production of granzyme B and IFN-γ, indicating compromised T cell priming. These findings suggest that alcohol-induced DC dysfunction may undermine the foundational steps of immune activation required for effective ICI therapy.

The gut microbiota also plays a pivotal role in modulating the immune landscape in alcohol-consuming individuals. Alcohol-induced dysbiosis—characterized by reduced microbial diversity and loss of beneficial taxa such as *Akkermansia muciniphila* and *Faecalibacterium prausnitzii*—has been shown to impair ICB efficacy by reducing dendritic cell maturation and T cell priming [[87], [88], [89], [90]]. Fecal microbiota transplantation (FMT) studies have demonstrated that restoring a favorable microbial profile can enhance ICB responses, suggesting that microbiome-targeted interventions may help overcome alcohol-related immunotherapy resistance. A large retrospective cohort study [91] evaluated the clinical impact of alcohol dependence on ICI therapy outcomes across multiple cancer types. While molecular data suggest that alcohol may upregulate PD-L1 expression and inhibit T cell function, the study found no statistically significant difference in overall survival between alcohol-dependent and non-dependent patients treated with ICIs. This discrepancy highlights the complexity of translating molecular mechanisms into clinical outcomes and underscores the need for stratified analyses considering alcohol exposure levels, duration, and immune status.

**Fig. 4:**
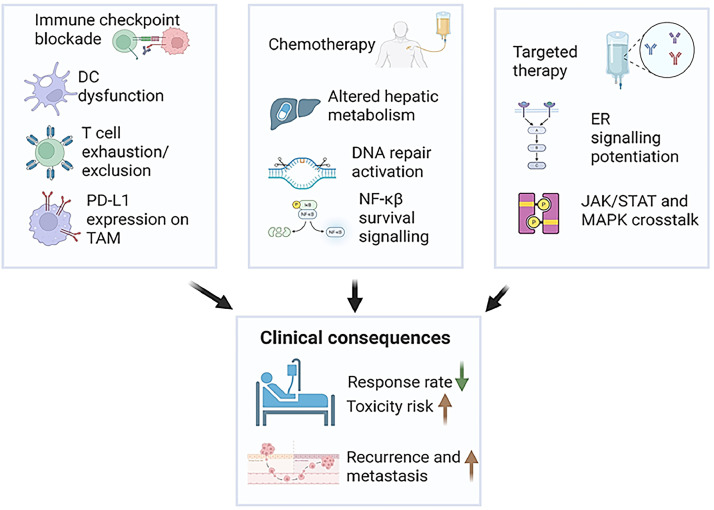
Models of therapy resistance exacerbated by alcohol exposure. Alcohol-induced changes in immune regulation hepatic metabolism and signaling pathways undermine the effectiveness of immune checkpoint blockade, chemotherapy and targeted therapies, leading to lower response rates, greater toxicity and increased recurrence.

Finally, chronic alcohol exposure promotes the expansion of immunosuppressive cell populations such as myeloid-derived suppressor cells (MDSCs) [92] and tumor-associated macrophages (TAMs) [93,94], which inhibit T cell responses and facilitate immune evasion [95]. MDSCs are derived from aberrant differentiation of myeloid progenitors under pathological conditions like chronic inflammation and cancer, both of which are exacerbated by alcohol consumption. These cells suppress T cell activity through mechanisms involving arginase-1, inducible nitric oxide synthase (iNOS), reactive oxygen species (ROS), and immunosuppressive cytokines such as IL-10 and TGF-β [96,97]. Alcohol-induced inflammation and oxidative stress can stimulate emergency myelopoiesis and skew hematopoietic differentiation toward MDSC lineages, thereby enhancing their immunosuppressive activity (Fig. 4). Fig. 4 synthesizes alcohol-induced myeloid skewing, including expansion of myeloid-derived suppressor cells and M2-like tumor-associated macrophages, with checkpoint dysregulation and suppressed T-cell function to illustrate core mechanisms underlying immunotherapy resistance.

Similarly, TAMs—particularly the M2-like phenotype—are recruited and polarized within the tumor microenvironment under the influence of alcohol-related cytokine shifts, including elevated IL-4, IL-10, and TGF-β. These macrophages support tumor growth, suppress cytotoxic immune responses, and contribute to stromal remodeling and metastasis [98,99]. TAMs also express immune checkpoint molecules such as PD-L1, TIM-3, and VISTA, further contributing to resistance against immune checkpoint blockade therapies. Collectively, these findings underscore the multifaceted impact of alcohol on immune regulation and highlight the need for alcohol cessation strategies in patients undergoing immunotherapy. Understanding these mechanisms may inform the development of adjunctive therapies to restore immune competence and improve treatment outcomes in alcohol-consuming cancer patients.

#### Alcohol induces chemotherapy resistance

In addition to compromising immunotherapy response through immune dysfunction, alcohol simultaneously affects the pharmacologic landscape of cancer treatment. The following section extends these concepts by addressing how alcohol-induced hepatic dysfunction, metabolic alterations, and genotoxic stress also impair chemotherapy metabolism and therapeutic response.

**Fig. 5:**
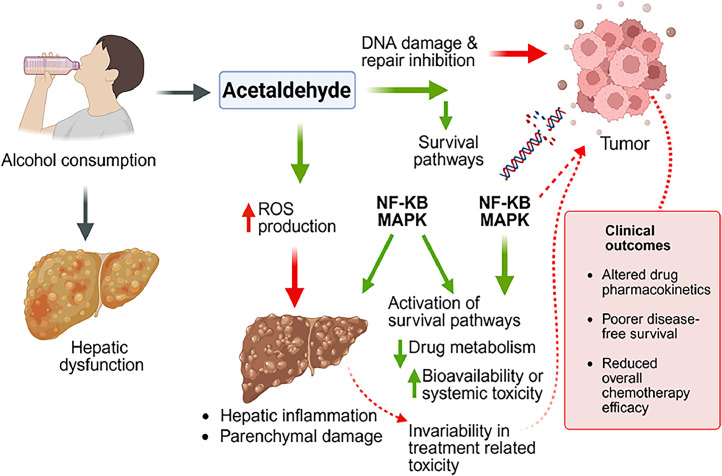
Alcohol-driven mechanisms that promote chemotherapy resistance. Alcohol metabolism generates acetaldehyde and oxidative stress that damage DNA and activate survival pathways, reducing tumor sensitivity to chemotherapy. Concurrent alcohol-induced liver dysfunction alters drug metabolism, leading to poorer treatment efficacy and outcomes.

Alcohol metabolism significantly influences chemotherapy outcomes by altering drug pharmacokinetics and cellular responses. Ethanol is primarily metabolized in the liver by alcohol dehydrogenase (ADH) and cytochrome P450 2E1 (CYP2E1), producing acetaldehyde—a reactive and genotoxic metabolite. Acetaldehyde can form DNA adducts and interfere with DNA repair mechanisms, particularly base excision repair and homologous recombination, thereby contributing to genomic instability and resistance to DNA-damaging chemotherapeutic agents [100]. This genotoxic stress may also activate survival pathways such as NF-κB and MAPK, which promote cell proliferation and inhibit apoptosis, further reducing chemotherapy efficacy [101]. Moreover, chronic alcohol consumption impairs liver function, which is central to drug metabolism and clearance. Damage to hepatic parenchyma, as seen in alcoholic liver disease, can alter the activity of drug-metabolizing enzymes and transporters, leading to suboptimal drug concentrations or increased toxicity [102,103]. These hepatic changes may affect the bioavailability of chemotherapeutic agents like cisplatin, doxorubicin, and 5-fluorouracil, compromising their therapeutic window. A meta-analysis evaluating alcohol use during chemotherapy and radiotherapy reported significantly poorer disease-free survival among alcohol-consuming patients (pooled HR: 2.05), although the impact on treatment-related toxicity varied across studies [104]. These findings collectively suggest that alcohol not only alters tumor biology but also disrupts systemic pharmacological responses, reinforcing the need for alcohol screening and tailored therapeutic strategies in oncology (Fig. 5). Fig. 5 integrates alcohol‑associated hepatic dysfunction, altered drug metabolism, and genotoxic stress to illustrate how systemic and tumor‑intrinsic mechanisms converge to impair chemotherapy efficacy.

Given these alcohol‑related disruptions in drug metabolism and intracellular trafficking, emerging precision‑delivery systems may help overcome such chemotherapy resistance. Lysosome‑targeted theranostic platforms—such as Zn(II)‑Schiff base complexes—enable controlled, lysosome‑specific drug release with real‑time imaging; however, these platforms remain investigational. While they show promises for improving intracellular drug exposure in preclinical settings, their ability to mitigate alcohol‑associated chemoresistance in humans has not been demonstrated and warrants clinical evaluation [105].

## Aggravating factors of alcohol-related cancer progression

Since alcohol-induced biological changes already increase cancer aggressiveness and treatment failure, understanding synergistic lifestyle and metabolic factors becomes essential. Accordingly, this section expands the framework by integrating smoking, obesity, metabolic disease, and demographic variables that further intensify alcohol-related cancer progression.

Smoking and alcohol consumption are interrelated behaviors that significantly impact public health, both individually and synergistically. Lauridsen et al., in a longitudinal cohort study [106] examined the impact of smoking and alcohol use in bladder cancer patients undergoing radical cystectomy. While a perioperative cessation intervention temporarily improved quit rates, it did not significantly enhance health-related quality of life. However, patients experiencing multiple complications had notably poorer outcomes, emphasizing the importance of preoperative risk reduction. In their broader cohort analysis, Lauridsen and colleagues also found that smoking and daily alcohol consumption substantially increased healthcare costs—13.8% higher for male smokers and 18.6% for female smokers, with alcohol-related costs rising 21.4% for males and 31.8% for females—highlighting not only the clinical but also the economic burden of these lifestyle factors. Similarly, Pelucchi et al. [107] reviewed the combined impact of alcohol and tobacco use on cancer risk, focusing on the upper aero-digestive tract and liver. They found that both substances independently increased the risk of cancers in the oral cavity, pharynx, larynx, and esophagus, but when used together, they had a multiplicative effect, significantly amplifying the risk. The region’s most directly exposed to alcohol and tobacco—such as the mouth and throat—were especially vulnerable. For esophageal squamous cell carcinoma, the combined use of alcohol and tobacco posed a particularly high risk. In the case of liver cancer, alcohol and tobacco acted as independent risk factors, each contributing separately to cancer development. These findings emphasized the need for integrated public health strategies to reduce exposure to both alcohol and tobacco.

Obesity and alcohol consumption are two significant modifiable lifestyle factors that have been independently associated with increased cancer risk. Recent studies have begun to explore their combined impact on cancer progression. Eroglu et al., have revealed that individuals with higher adiposity who consumed alcohol above recommended guidelines had a significantly elevated risk of developing both alcohol- and obesity-related cancers. Specifically, those in the highest body fat percentage tertile who drank above guidelines had a 61% increased risk of alcohol-related cancers compared to low-adiposity non-drinkers [108]. Similarly, Macdonald et al., in a systematic review synthesized data from 24 studies and found that while both alcohol and obesity independently contributed to cancer risk, postulating for a synergistic interaction between the two was inconsistent across cancer types [109]. Furthermore, Seppa et al., have estimated that one in ten malignant solid tumors could be attributed to the combined effects of excess body weight and alcohol consumption [110]. This study highlighted that in women, the joint contribution of these factors to cancer burden even surpassed that of smoking, underscoring the growing public health relevance of obesity and alcohol-related interventions.

Similarly, Ginsburg et al. [111] reported that hyperinsulinemia promotes tumor cell proliferation, survival, and angiogenesis via activation of the PI3K/Akt and MAPK pathways. Insulin also enhances estrogen bioavailability by stimulating aromatase and suppressing sex hormone-binding globulin, thereby synergizing with estrogen to drive tumor progression. Furthermore, Hong et al. [112] showed that alcohol consumption sensitizes mammary tumor cells to both insulin and estrogen, amplifying their mitogenic effects and accelerating tumor growth and aggressiveness. Together, these studies emphasize the need for integrated public health strategies that address alcohol moderation as a key component in obesity prevention and management.

Alcohol consumption and nutrition are closely linked to cancer risk, particularly in individuals with existing lifestyle risk factors such as smoking. The Alpha-Tocopherol Beta-Carotene Cancer Prevention Study (ATBC) investigated the effects of vitamin E and β-carotene supplementation in male smokers and found that β-carotene increased lung cancer incidence, especially among heavy smokers [113]. Similarly, Albanes et al., showed no protective effect of these supplements on overall cancer risk and even suggested increased mortality with β-carotene use [114]. These findings underscore the complex interplay between dietary antioxidants, alcohol use, and smoking, suggesting that supplementation alone may not counteract the carcinogenic effects of these behaviors and may, in some cases, worsen outcomes.

On the other hand, physical activity and alcohol consumption are both influential lifestyle factors in cancer risk and survivorship, often interacting in complex ways. Mama et al. have reported that rural cancer survivors who were moderately or highly physically active had significantly higher odds of alcohol use, suggesting that these behaviors may co-occur even in populations at elevated cancer risk [115]. In contrast, a pooled analysis by Feng et al., of over 54,000 British adults revealed that while hazardous and harmful alcohol consumption significantly increased the risk of alcohol-related cancer mortality, engaging in adequate physical activity attenuated these risks, highlighting physical activity as a potential protective factor [116]. Supporting this, Esther Molina-Montes et al., showed that adherence to healthy lifestyle behaviors—including regular physical activity and limited alcohol intake—was associated with reduced cancer mortality, reinforcing the importance of integrated lifestyle interventions for cancer prevention [117].

Demographic and social determinants significantly shape alcohol consumption patterns and their implications for cancer risk. Gender differences are notable, with postmenopausal women being particularly vulnerable to alcohol-related breast cancer due to hormonal changes that affect alcohol metabolism [118]. Gender differences in alcohol consumption extend beyond frequency to include physiological, psychological, and oncological impacts. Men typically consume larger quantities of alcohol and are more prone to behavioral issues, injuries, and alcohol-related deaths. In contrast, women metabolize alcohol more slowly, resulting in higher blood alcohol concentrations per drink [2].This slower metabolism leads to prolonged exposure to acetaldehyde, a genotoxic metabolite that promotes DNA damage and cancer initiation [119,120]. Women also face distinct mental health risks, including elevated rates of depression and anxiety, which can exacerbate alcohol misuse [121]. Additionally, women are more susceptible to hangovers, memory blackouts, cardiovascular disease, and liver inflammation [2]. Importantly, alcohol elevates circulating estrogen levels, increasing the risk of hormone-sensitive cancers such as breast cancer [122]. In a cohort of 93,835 younger women from the Nurses’ Health Study II, alcohol consumption was not associated with breast cancer risk overall. However, among women with both a family history of breast cancer and low folate intake (<400 μg/day), alcohol consumption was linked to a significantly increased risk (HR = 1.82) [6]. These findings highlight that women tend to experience more severe physiological, psychological, and cancer-related consequences from alcohol consumption than men. Racial and ethnic disparities also influence outcomes; for example, African American and Hispanic women often report more severe menopausal symptoms and may face higher risks due to systemic barriers in healthcare access [123,124]. Additionally, Indigenous and multi-ethnic populations have been shown to experience greater symptom burden and reduced access to preventive care [124]. Geographic disparities, such as living in rural or underserved areas, further limit access to cancer screening and treatment services [125]. Socioeconomic status (SES) is another critical factor—lower SES is associated with earlier menopause, higher alcohol use, and increased cancer risk due to limited healthcare access and health literacy [126]. These intersecting factors highlight the need for culturally tailored and equitable public health strategies to mitigate alcohol-related cancer risks across diverse populations [127].

Alcohol consumption influences the pathophysiology of diabetes through multiple interconnected mechanisms affecting glucose metabolism, insulin sensitivity, oxidative stress, and pancreatic β-cell function. Ethanol metabolism in the liver alters the NAD⁺/NADH ratio, impairing gluconeogenesis and glycogenolysis—key processes for maintaining blood glucose levels—especially during fasting, thereby increasing the risk of hypoglycemia in diabetic individuals [128]. Chronic alcohol intake also disrupts hepatic glucose output and promotes hyperglycemia by inducing insulin resistance, particularly in individuals with type 2 diabetes [128]. Meta-analyses have shown that while moderate alcohol consumption may reduce fasting insulin and HbA1c levels in some populations, excessive intake worsens insulin sensitivity and glycemic control [129,130]. At the cellular level, alcohol impairs insulin signaling in glucoregulatory tissues such as skeletal muscle, liver, and adipose tissue, leading to reduced glucose uptake and increased circulating glucose levels [128,130]. Furthermore, alcohol-induced oxidative stress plays a central role in diabetes progression. It disrupts redox balance, increases reactive oxygen species (ROS), and activates inflammatory pathways that damage insulin-producing pancreatic β-cells [131]. These effects are compounded by alcohol’s direct toxicity to pancreatic cells, including acinar and ductal cells, and its metabolites such as acetaldehyde and fatty acid ethyl esters, which contribute to β-cell apoptosis and impaired insulin secretion [132].

Age plays a critical role in modulating the risk of cancer development among individuals who consume alcohol, acting both as a biological amplifier and a temporal marker of cumulative exposure. As individuals age, physiological changes such as reduced metabolic efficiency and impaired DNA repair mechanisms increase susceptibility to carcinogenic effects of alcohol. Moreover, long-term alcohol consumption often correlates with prolonged exposure to acetaldehyde, a toxic metabolite known to damage cellular structures and promote oncogenesis. Hur et al. [133], showed that Mendelian randomization techniques underscore that age-related genetic and metabolic profiles can exacerbate the impact of alcohol on cancer risk, particularly when confounding variables are properly accounted for. Similarly, according to the study by Zhang et al. [134], causal inference frameworks applied to observational health data reveal that age significantly interacts with lifestyle factors like alcohol use, influencing disease progression, and outcomes. These findings collectively suggest that older adults who consume alcohol are at a disproportionately higher risk for cancer, necessitating age-specific prevention and intervention strategies.

## Pharmacological interventions for alcohol use disorder (AUD)

Given the multifactorial ways alcohol worsens cancer risk and treatment response, addressing alcohol use disorder is a critical therapeutic priority. Thus, the next section summarizes current pharmacological strategies to reduce alcohol consumption and mitigate downstream oncogenic effects.

Pharmacological interventions for alcohol use disorder (AUD) have become essential components of comprehensive treatment strategies, with several medications targeting different aspects of addiction. Naltrexone [135], an opioid receptor antagonist, reduced the rewarding effects of alcohol and helped diminish cravings. It was available in both oral and extended-release injectable forms and showed improved outcomes when initiated during hospitalization, especially when combined with behavioral therapies[136]. Acamprosate [137,138] worked by modulating glutamatergic neurotransmission, helping to restore the excitatory-inhibitory balance in the brain disrupted by chronic alcohol use. It was particularly effective in maintaining abstinence among individuals who had already stopped drinking and was well-tolerated, though it did not directly reduce cravings. Disulfiram [139,140], a deterrent agent, inhibited acetaldehyde dehydrogenase, causing unpleasant reactions such as nausea and palpitations upon alcohol intake. While it did not address cravings or withdrawal symptoms, Disulfiram was useful for individuals committed to abstinence and showed better efficacy when administered under supervision as part of a structured treatment plan. Each of these medications offered unique benefits and worked best when integrated with psychosocial support, counseling, and behavioral therapy. In summary, Naltrexone, Acamprosate, and Disulfiram offer complementary approaches to treating alcohol use disorders, targeting cravings, neurochemical balance, and behavioral deterrence, and are most effective when integrated with psychosocial support.

## Public health recommendations

Building upon mechanistic, immunological, and treatment focused insights, this section translates the biological evidence into actionable public health strategies aimed at reducing alcohol-associated cancer burden. Public health strategies must adopt a multifaceted approach to mitigate alcohol-related harm. These recommendations align with international guidance that emphasizes risk from any level of alcohol exposure and prioritizes population-level reduction strategies; local implementation should adapt to healthcare access, cultural context, and competing risks.

First, policies should promote awareness of alcohol’s role in cancer and chronic disease risk, countering the misconception of its safety in moderation. Second, itemized interventions targeting both alcohol and co-risk behaviors such as smoking and poor diet are critical, given their synergistic effects. Third, tailored guidelines for vulnerable populations—including women, individuals with diabetes or hypertension, and low socioeconomic groups—can reduce disparities. Fourth, preventive measures should also emphasize moderation, early screening for alcohol-related diseases, and lifestyle modifications like exercise and dietary improvements to offset alcohol’s metabolic and immune effects. Finally, expanding access to treatment for AUD through pharmacological and behavioral therapies remains essential to reduce disease burden and healthcare costs. These recommendations are consistent with international guidance from the World Health Organization (WHO) and the International Agency for Research on Cancer (IARC), which classify alcohol consumption as a Group 1 carcinogen and emphasize that no safe level of alcohol intake for cancer prevention has been established (IARC Monographs 100E; WHO Global Alcohol Action Plan 2022–2030). Translating these recommendations into practice requires population level alcohol-reduction policies, routine screening and counseling for alcohol use within primary care and oncology settings, and integration of alcohol-reduction strategies into existing cancer prevention, smoking cessation, and obesity-management programs [141,142]. Taken together, these actions provide actionable pathways for translating global guidance into routine clinical practice and public health policy.

## Conclusions and future directions and scope

Unlike prior reviews that largely focus on epidemiologic associations or isolated carcinogenic mechanisms, this review offers an integrated mechanistic framework linking alcohol exposure to tumor immune microenvironment (TIME) remodeling and therapeutic resistance across immunotherapy, targeted therapies, and chemotherapy. By connecting genotoxic, metabolic, inflammatory, immune, microbiome, and exosome mediated pathways, we advance a system-level understanding of how alcohol drives immune dysfunction and treatment failure, with direct translational implications.

While cross-sectional and case–control studies have provided strong evidence linking alcohol consumption to cancer and immune dysfunction, they fall short in capturing the temporal and causal dynamics of disease development. Most available data are retrospective, which limits the ability to establish directionality between alcohol exposure and biological changes such as immune dysregulation, oxidative stress, and epigenetic modifications. This gap is critical because these processes unfold over years, and short-term snapshots cannot fully explain how cumulative alcohol exposure interacts with genetic predisposition and environmental factors to drive cancer risk.

To address these limitations, future research must prioritize large-scale longitudinal studies that follow individuals over extended periods, integrating behavioral data with molecular and clinical endpoints. These studies should incorporate multi-omics profiling—including genomics, epigenomics, metabolomics, and proteomics—alongside immune and inflammatory biomarkers to unravel causal pathways. Advanced imaging and real-time biospecimen analysis will be essential for monitoring tumor microenvironment changes and immune cell dynamics in response to sustained alcohol exposure. Harmonizing these cohorts across diverse populations will clarify dose–response relationships, age-related vulnerabilities, and gene–environment interactions. Such approaches will not only strengthen causal inference but also inform precision prevention strategies and targeted interventions for alcohol-related cancer risk.

Animal models have significantly advanced our understanding of how chronic alcohol exposure influences cancer development, revealing its role as a tumor promoter rather than a direct carcinogen. Rodent models dominate this research due to their accessibility and genetic flexibility, yet they fall short in replicating long-term alcohol exposure and aging-related cancer risk, limiting translational relevance. Physiological differences between rodents and humans further constrain applicability to clinical settings. To overcome these challenges, future research should incorporate non-human primate models that better mimic human metabolism and allow extended observation periods. Integrating advanced imaging, multi-omics approaches, and standardized dosing protocols will enhance reproducibility and mechanistic insights. Ultimately, combining chronic alcohol exposure studies with genetic and environmental risk factors will provide a more comprehensive framework for understanding and mitigating alcohol-related cancer progression.

Mechanistic studies remain a priority, particularly those focusing on immune cell signaling, cytokine modulation, and tumor microenvironment changes. While oxidative damage and inflammatory signaling pathways are well-documented, the precise sequence of immune alterations leading to therapy resistance remains unclear. The synergistic impact of alcohol and smoking also warrants deeper investigation, as alcohol may enhance the bioavailability or toxicity of carcinogenic compounds in tobacco, amplifying cancer risk.

Despite growing interest in lifestyle interventions, the molecular mechanisms by which diet protects against alcohol-related liver disease and how exercise mitigates depressive symptoms associated with alcohol consumption remain poorly understood. These gaps highlight the need for integrative studies combining behavioral, metabolic, and molecular data. Human and preclinical models, supported by robust clinical trials, are essential to translating these mechanistic insights into practical interventions. Longitudinal studies should explore how sustained alcohol consumption interacts with dietary patterns and exercise to influence cancer risk and overall health outcomes. Identifying which diets—such as Mediterranean, plant-based, or antioxidant-rich approaches—offer the greatest protection against alcohol-related liver disease and systemic inflammation will be critical. Similarly, trials combining behavioral interventions with molecular profiling can clarify how exercise mitigates depressive symptoms and modulates immune and metabolic pathways in individuals with alcohol use disorder. These integrative, long-term studies will provide a foundation for personalized prevention strategies and targeted therapies.

More detailed mechanistic insights from animal models, multi-omics profiling, and human biospecimen studies will help clarify how chronic alcohol exposure drives cancer initiation, progression, and treatment outcomes. Importantly, future research should also explore disproportionate health risks among certain population groups, moving beyond socioeconomic explanations to investigate molecular and genetic contributors to vulnerability. To advance the field, future studies must build established evidence while addressing critical gaps. Current research confirms that chronic alcohol consumption is a modifiable risk factor for cancer, acting through mechanisms such as acetaldehyde-induced DNA damage, oxidative stress, hormonal dysregulation, and immune suppression. Where treatment responses are discussed, these interpretations reflect mechanistic and preclinical evidence more than confirmed clinical impact, and substantial uncertainty remains due to the predominance of observational human data.

These pathways not only initiate carcinogenesis but also contribute to therapy resistance and metastatic potential. However, major gaps remain in understanding the precise molecular sequence linking alcohol exposure to immune dysfunction and treatment outcomes. Similarly, the combined impact of alcohol and smoking on carcinogen bioavailability and toxicity requires deeper mechanistic exploration. Clinical trials should examine how alcohol use influences the efficacy of chemotherapy, immunotherapy, and targeted treatments, enabling personalized interventions. At the public health level, refining global guidelines to incorporate genetic, metabolic, and lifestyle variability will improve risk stratification. Expanding the clinical utility of exosomal miRNAs, cytokines, and metabolic markers could transform early detection and prognostic assessment. Finally, prevention strategies must integrate alcohol reduction with obesity, smoking cessation, and nutrition programs addressing interconnected risks holistically. These efforts will require collaboration across epidemiology, molecular biology, behavioral science, and health policy to close the translational gap and reduce alcohol-related cancer burden.

## Declarations

**Declaration of generative AI use:** Generative AI (Copilot) was used minimally for grammar and language corrections.

**Ethics approval and consent to participate:** Not applicable.

**Consent for publication:** All authors have read and approved the final version of the manuscript.

**Availability of data and materials:** Not applicable.

## Funding

This work was supported by the National Institutes of Health National Cancer Institute grants R01CA205067 and by the Department of Defense W81XWH2110075 (to KW). The figures were created in BioRender (https://www.biorender.com/).

## CRediT authorship contribution statement

**Ravindra Pramod Deshpande:** Writing – original draft, Data curation. **Abhishek Tyagi:** Writing – review & editing, Data curation. **Kounosuke Watabe:** Writing – review & editing, Supervision, Funding acquisition, Conceptualization.

## Declaration of competing interest

All authors declare that they have no known competing financial interests or personal relationships that could have appeared to influence the work reported in this paper.
